# Parallel Multi-Deque Partition Dual-Deque Merge sorting algorithm using OpenMP

**DOI:** 10.1038/s41598-023-33583-4

**Published:** 2023-04-19

**Authors:** Sirilak Ketchaya, Apisit Rattanatranurak

**Affiliations:** 1 Faculty of Science and Technology, Suan Sunandha Rajabhat University, Bangkok, Thailand; 2 Department of Computer and Information Sciences, Faculty of Applied Science, King Mongkut’s University of Technology North Bangkok, Bangkok, Thailand

**Keywords:** Computer science, Software

## Abstract

Quicksort is an important algorithm that uses the divide and conquer concept, and it can be run to solve any problem. The performance of the algorithm can be improved by implementing this algorithm in parallel. In this paper, the parallel sorting algorithm named the Multi-Deque Partition Dual-Deque Merge Sorting algorithm (*MPDMSort*) is proposed and run on a shared memory system. This algorithm contains the *Multi-Deque Partitioning phase*, which is a block-based parallel partitioning algorithm, and the *Dual-Deque Merging phase*, which is a merging algorithm without compare-and-swap operations and sorts the small data with the sorting function of the standard template library. The OpenMP library, which is an application programming interface used to develop the parallel implementation of this algorithm, is implemented in *MPDMSort*. Two computers (one with an Intel Xeon Gold 6142 CPU and the other with an Intel Core i7-11700 CPU) running Ubuntu Linux are used in this experiment. The results show that *MPDMSort* is faster than parallel balanced quicksort and multiway merge sort on the large random distribution data. A speedup of 13.81$\times$ and speedup per thread of 0.86 can be obtained. Thus, developers can use these parallel partitioning and merging algorithms to improve the performance of related algorithms.

## Introduction

Sorting is a well-known algorithm that can be implemented in other algorithms to solve biological, scientific, engineering, and big data problems. The popular sorting algorithm is Quicksort^1^. It is an important algorithm that uses the divide-and-conquer concept to sort the data. It partitions the data into smaller sizes (divide) and then sorts those data (conquer). Basically, there are 2 steps in the Quicksort algorithm. The first step is partitioning, which divides the data using their pivot recursively into smaller sizes. This step runs until the data are smaller than the algorithm’s cutoff size. Finally, the sorting step is executed to sort the data.

There are several works study in parallel sorting algorithm on multi-core CPU^2–6^, and branch misprediction and cache misses in parallel sorting algorithm^7–10^. It should avoid branch misprediction and reduce cache misses by improving locality while develop parallel sorting algorithm on multi-core CPU. These concepts are used to design our parallel sorting algorithm to improve its performance.

In this paper, we propose the parallel *Multi-Deque Partition Dual-Deque Merge Sorting* algorithm, which consists of three phases: the *Multi-Deque Partitioning phase*, which is block-based parallel partitioning. Each thread contains a double-ended queue (deque), which keeps the boundaries of each block. This phase partitions the data of each block and pushes the new boundaries of the partitioned data in the dual deque. Then, the *Dual-Deque Merging phase* is executed to merge data into the correct positions using the new boundaries of the dual deque without compare-and-swap operations. These two phases are recursively executed until the data are sufficiently small. Finally, the *Sorting phase* is executed to sort the data independently.

This work uses the *OpenMP* library^11^ to execute the parallel sorting algorithm. There are several metrics to measure the performance of this algorithm and compare it with the sequential sorting algorithm, such as Run time, Speedup, and Speedup per thread. Moreover, we switch *Hoare’s partitioning*^12^ to *Lomuto’s partitioning* algorithm in the *Multi-Deque Partitioning phase*. Finally, the *Perf profiling tool*^13^ is run to measure the metrics to analyze the performance of this algorithm.

Our proposed parallel sorting algorithm uses the block-based partitioning concept which has the problem while merging the data in each block. Most of solutions compare and swap the leftover data to the middle of the array. Then, they use the sequential or parallel partitioning algorithm to partition it again. In this paper, *Dual-Deque Merging* phase is proposed as the main contribution to solve this problem to reduce compare-and-swap operations in the algorithm which consume run time.

In this paper, the contributions are as follows: (1) the parallel sorting algorithm called the *Multi-Deque Partition Dual-Deque Merge Sorting* algorithm (*MPDMSort*), which contains *Multi-Deque Partitioning*, *Dual-Deque Merging*, and *Sorting phases*, is proposed. (2) The partitioning algorithms in the *Multi-Deque Partitioning phase*, such as *Hoare’s* and *Lomuto’s partitioning* algorithms, are compared. (3) Run time, Speedup, Speedup/core and thread, and other metrics that can be measured from the *Perf profiling tool* of *MPDMSort*, *Parallel Balanced Quicksort*, and *Multiway merge sort* are compared and analyzed. We organized this paper as follows: Background and related work are shown in section “Background and related work”. The *Multi-Deque Partition Dual-Deque Merge Sorting* algorithm is proposed in section “Multi-Deque Partition Dual-Deque Merge Sorting algorithm”. Section “Experiments, results and discussions” shows the experiments, results, and discussions of parallel sorting algorithms. Finally, the conclusion and future work are shown in section “Conclusions”.

## Background and related work

This section introduces a sequential standard sorting algorithm called *STLSort* and parallel standard sorting algorithms called *Parallel Balanced Quicksort* (*BQSort*) and *Multiway merge sort* (*MWSort*). Finally, we proposed and compared related parallel sorting algorithms.

### Sequential and parallel standard sorting algorithms

There is a sorting standard library function that can sequentially sort the data. *STLSort*^14^ is an important sorting function in the C++ language. Developers can implement this function by declaring $<algorithm>$ directive. It contains the *Introsort* algorithm, which consists of quicksort and heapsort. While the data are sufficiently small, the insertion sort is executed to sort those data.

There are two standard sorting algorithms in parallel mode. *Parallel Balanced Quicksort* (*BQSort*)^15^ is the parallel sorting algorithm. It uses block-based partitioning concepts such as Tsigas and Zhang’s algorithm^16^. Each block runs a compare-and-swap operation and swaps all leftover to the middle of those data. Then, the sequential partitioning algorithm is executed. *Multiway merge sort* (*MWSort*) separates data equally and sorts them independently. Then, a parallel multiway merge algorithm is called to merge the data in parallel. Note that it requires an array that is used to store the temporary data. This algorithm is more stable than the quicksort algorithm.

### Related parallel sorting algorithms

The performance of many quicksort algorithms is improved by parallel algorithm techniques, which can be executed on shared memory systems. The parallel quicksort algorithm concept begins with partitioning the data in parallel. Next, the partitioned data are merged. While the data are smaller, they are sorted by any sorting algorithm independently.

In 1990, a parallel quicksort algorithm on an ideal parallel random access machine using Fetch-and-Add instruction was proposed^17^. The speedup of this parallel quicksort is up to 400$\times$ on 500 processors when sorting $2^{20}$ data. Tsigas and Zhang^16^ proposed *PQuicksort* in 2003. This sorting algorithm divides the data into blocks and neutralizes them in parallel. A speedup of 11$\times$ with a 32-core processor can be obtained. In 2004, the implementation of parallel quicksorting using *pthreads* and *OpenMP 2.0* was presented^18^. A multicore standard template library was proposed^19^, which contains a parallel sorting algorithm that is similar to Tsigas and Zhang’s concept^16^. A speedup of 3.24$\times$ is achieved on a 4-core processor. Then, a parallel introspective sorting algorithm using a deque-free work-stealing technique was proposed^20^. In 2009, *Multisort*, which is a parallel quicksort algorithm that partitions data, sorts them independently using quicksort and merges them in parallel, was presented^21^. A speedup of 13.6$\times$ is achieved when sorting data using this algorithm on a 32-core processor. Man et al.^22^ proposed a parallel sorting algorithm named *psort*. It divides the data into several groups and sorts them locally in parallel. Then, those sorted groups of data are merged and finally sorted sequentially. Its speedup is up to 11$\times$ on a 24-core processor. Meanwhile, *Parallel Introspective quicksort* was developed and run on an embedded OMAP-4430, which consists of a dual core processor^23^. Its speedup is up to 1.47$\times$. Mahafzah^24^ shows a parallel sorting algorithm with a multipivot concept that partitions the data up to 8 threads. Its speedup is up to 3.8$\times$ on the 2-core with hyperthreading technology processor. In 2016, *Parallel Partition and Merge Quick sort* (*PPMQsort*) was proposed^25^. A speedup of 12.29$\times$ with 8 cores with the hyperthreading technology Xeon E5520 can be obtained. Taotiamton and Kittitornkun^26^ presented the parallel *Hybrid Dual Pivot Sort* (*HDPSort*) in 2017. Its partitioning function uses two pivots to partition data. Moreover, *Lomuto’s* and *Hoare’s partitioning* algorithms are implemented, and the performance is compared. Speedups of 3.02$\times$ and 2.49$\times$ can be obtained on AMD FX-8320 and Intel Core i7-2600 machines, respectively. In the same year, Marszałek^27^ introduced Parallel Modified Merge Sort Algorithm based on Parallel Random Access Machine. It was proved that it can sort *n* elements in the maximum time $2n-\log {2}{n}-2$. One year later, Marszałek et al.^28^ proposed a fully flexible parallel merge sort algorithm which the computational complexity is optimized. The operational time is equal to $O(\sqrt{N})$ and it is flexible to an increasing number of processor cores.

In 2020, a block-based sorting algorithm named the *MultiStack Parallel Partition Sorting* algorithm (*MSPSort*) was presented^29^^30^. Each thread uses left and right stacks to keep the boundaries of each block. It partitions from the leftmost and rightmost of the array to the middle of the array. Its run time is better than those of *BQSort* and *MWSort* on the Intel i7-2600, AMD R7-1700 and R9-2920 processors. Moreover, a parallel quicksort algorithm for OTIS-HHC optoelectronic architecture was proposed by Al-Adwan et al.^31^. Its average effeciency on 1,152 processors is up to 0.72. Langr and Schovánková^32^ developed a multithreaded quicksort named *CPP11sort*. A parallel speedup of 44.2$\times$ can be obtained on the 56-core server and 14.5$\times$ on the 10-core Hyperthread machine.

Recently, the *Dual Parallel Partition Sorting* algorithm (*DPPSort*)^33^ was proposed in 2022. It divides data into two parts and partitions them independently in parallel using *OpenMP*. Then, the *Multi-Swap* function is called to merge the data without the compare-and-swap operation. A speedup of 6.82$\times$ can be obtained on a 4-core Hyperthread Intel i7-6770 machine.

There are several methods to improve the performance of sorting algorithms. Marowka^34^ investigated sorting algorithm proposed by Cormen^35^ which is vector-based quicksort algorithm. This work is implemented using process-based and thread-based models. However, it did not exhibit good scalability because of its overhead. Gebali et al.^36^ proposed a *Parallel Multidimensional Lookahead Sorting* algorithm which is suitable for GPGPU and massively parallel processor systems. Cortis et al.^37^ developed *Parallelised Modified Quickselect* algorithm which used the same concept of quicksort algorithm. Then, this algorithm was implemented into their parallel quicksort algorithm. The results showed that their algorithm is faster than the original quicksort. Helal and Shaheen^38^ enhanced *iHmas* algorithm which is parallel partitioning and sorting algorithm using MPI^39^. The bottleneck and single point of failure are reduced compared with their *Hams* algorithm. Mubarak et al.^40^ proposed a preprocessing techniques before run on any sorting algorithm. Insertion sort and quicksort are run using these methods. The time complexities of the proposed sorting algorithms are reduced.

## Multi-Deque Partition Dual-Deque Merge Sorting algorithm

A sorting algorithm named the *Multi-Deque Partition Dual-Deque Merge sorting* algorithm (*MPDMSort*) is proposed in this section. In this work, it comprises 5 algorithms. First, the *Multi-Deque Partition Dual-Deque Merge Sort* function, *MPDMSort*, is the main function for partitioning and sorting, as shown in Algorithm 1. The *median of five* function (*MedianOf5*) is the algorithm for selecting pivot (Algorithm 2). The *InitBlocks* function (Algorithm 3) is the block initialization algorithm that appends the boundary of each block into a double-ended queue (deque). The *MPPDMPar* function (Algorithm 4) is the parallel partitioning function, which is called the *Multi-Deque Partitioning phase*. *DualDeqMerge* or *Dual-Deque Merging phase* (Algorithm 5) is a merging function used to merge the data from the *MPDMPar* function.

### Multi-Deque partitioning phase

It begins with selecting the pivot to divide the data into smaller subarrays. The *median of five* algorithm which is the pivot selection algorithm, is called (Line 6, Algorithm 1). Then, the parallel partitioning function (*MPDMPar*) is executed and returns a new pivot position (Line 6, Algorithm 1). The *MPDMSort* is called recursively with the divide and conquer concept (Lines 8 and 10, Algorithm 1) in parallel using the **omp task** (Lines 7 and 9, Algorithm 1).

The *median of five* function or *MedianOf5* (Line 5, Algorithm 1) is the pivot selection function. It selects the *mid* position by calculating the left and right positions of that subarray (Line 1, Algorithm 1). The positions of quarter (*qt*1) and third-quarter (*qt*3) are calculated using the *left*, *mid*, and *right* positions (Lines 2 and 3, Algorithm 2). Then, the data of *left*, *qt*1, *mid*, *qt*3, and *right* are sorted (Line 4, Algorithm 2). Finally, the *mid* position is returned to *MPDMSort*.
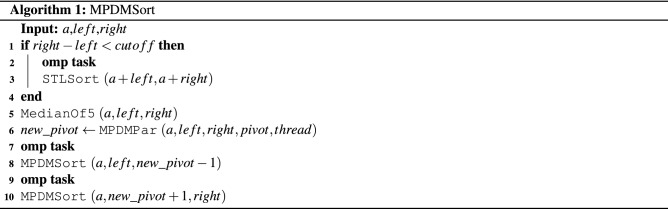

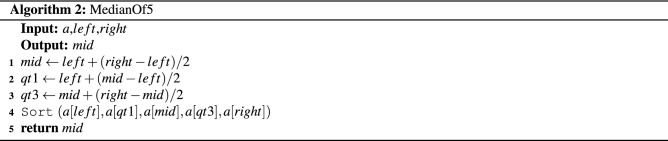


In this paper, *MPDMPar* is the parallel partitioning algorithm in this paper. It begins with swapping the pivot and left data of that subarray (Line 1, Algorithm 3). Then, the *InitBlocks* function is executed to initial multiple blocks using a double-ended queue (deque) with *Blocksize* size (Line 2, Algorithm 3). Note that this function returns *deq*, which keeps the boundary of blocks that can be used to partition the data in each block. After that, *Hoare’s partitioning* in block is executed using **omp parallel for** (Lines 5–6, Algorithm 3). Every *deq* in each thread is popped for the left *i* and right *j* boundaries in the critical section (Lines 8–9, Algorithm 3). Note that the left and right boundaries that pop from *deq* are assigned to *temp* and *temp*2, respectively (Line 10, Algorithm 3). Moreover, *Hoare’s partitioning* algorithm in each block is run in parallel (Lines 11–22, Algorithm 3). After that, the partitioned boundaries are pushed to dual-deque. The first deque *dl* keeps the boundaries of data that are less than or equal to pivot (Line 25, Algorithm 3). The second *dg* keeps the boundaries of data that are greater than pivot (Line 26, Algorithm 3). Then, both *dl* and *dg* are passed to the *DualDeqMerge* function to merge the data without the compare-and-swap operation (Line 29, Algorithm 3) and return the leftover boundary to run sequential partitioning (Line 30, Algorithm 3). Finally, the $new\_pivot$ from sequential partitioning and data at the left position of that subarray are swapped and then returned (Lines 31–32, Algorithm 3).
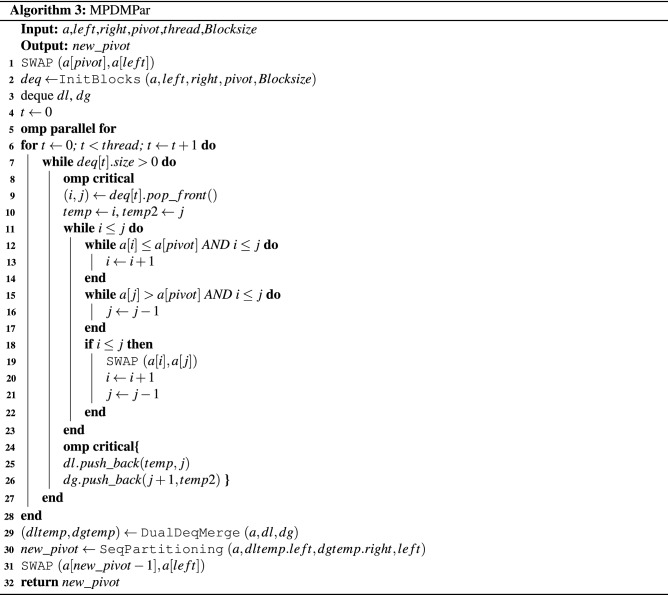


In this paper, the multideque data structures are created before parallel partitioning. It keeps the boundaries of each block. First, the number of blocks is calculated (Lines 1–2, Algorithm 4). The multideque array *deq* is created with *thread* length (Line 3, Algorithm 4), and then the boundaries are pushed into each *deq* (Lines 5–8, Algorithm 4). Note that if there is remaining block, the last boundaries of block will be pushed into the *deq* (Lines 9–12, Algorithm 4).
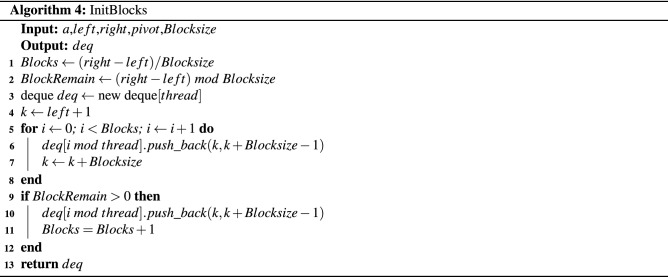


### Dual-Deque merging phase

The previous phase shows the block-based parallel partitioning concept using multideque as its data structure to keep the block boundaries. It provides the data that are less than or equal to pivot and greater than pivot in each block. Therefore, we push the boundaries of data that are less than or equal to pivot value and greater than pivot value in *dl* and *dg* deques, respectively. The data will be merged in this phase without the compare-and-swap operation.
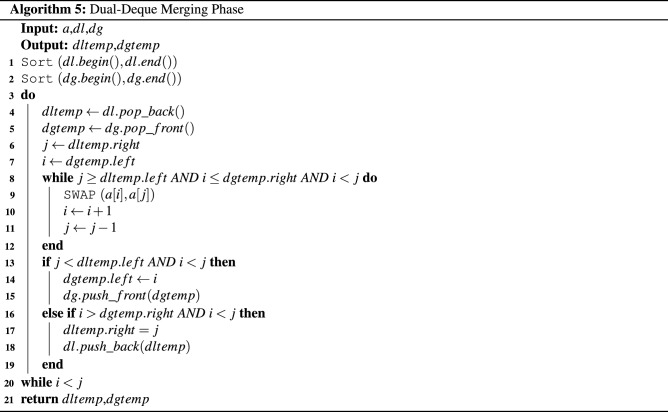


**Figure 1:**
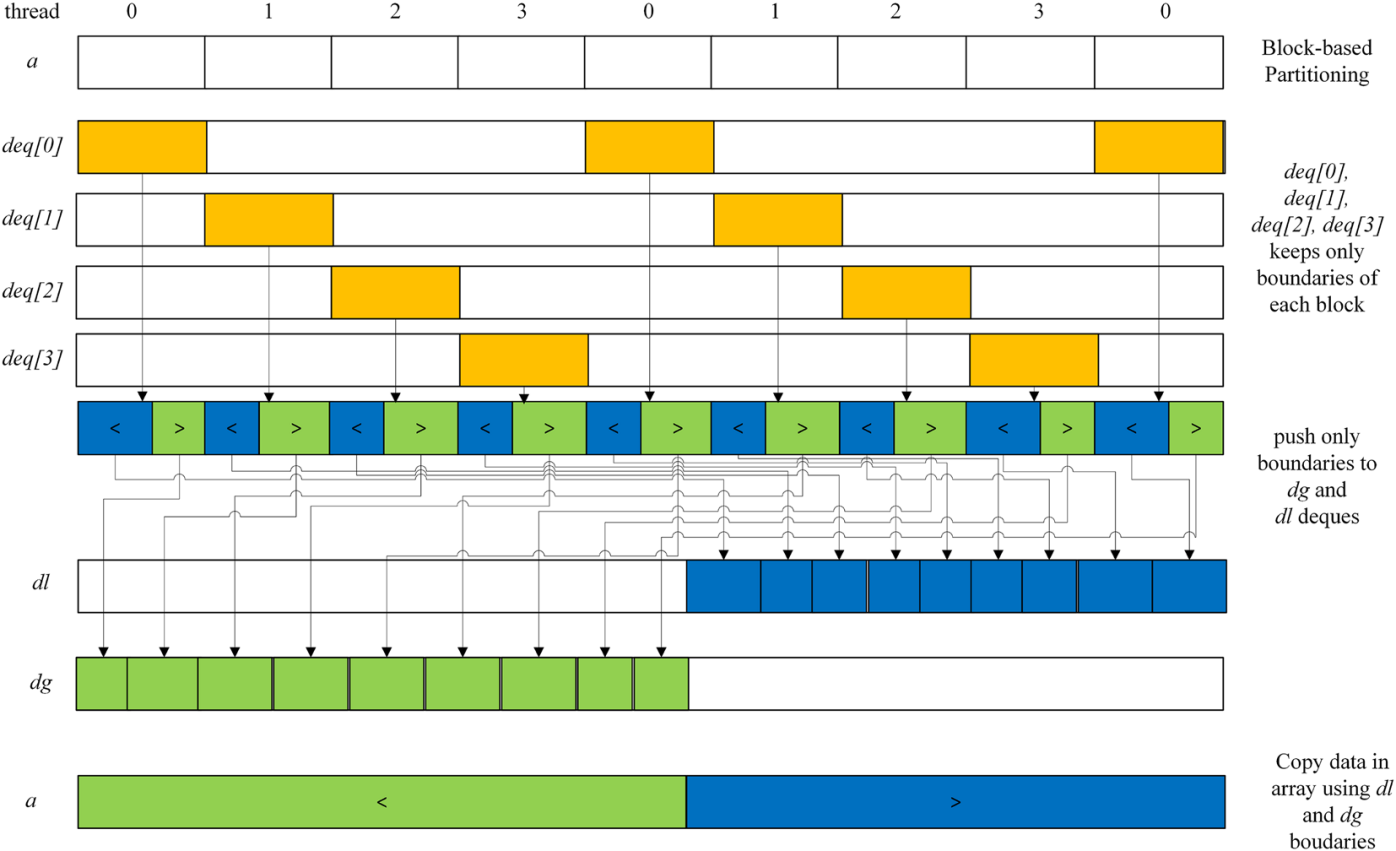
The illustration of the Multi-Deque Partitioning Phase and Dual-Deque Merging Phase.

The *dl* and *dg* deques keep the boundaries of data in the *Multi-Deque Partitioning phase*. Therefore, the boundaries are not sorted. First, *dl* and *dg* are sorted before merging the data (Lines 1–2, Algorithm 5). Then, *j* is popped back from *dl*, and *i* is popped front from *dg*. *j* is an index used to swap the data that are less than or equal to pivot value in the right block of the array. *i* is an index used to swap the data that are greater than pivot in the left block of the array. The data of the left and right blocks are swapped, where *j* is greater than or equal to the boundary of its block and *i* is less than or equal to the boundary of its block (Lines 8–12, Algorithm 5). If there are leftover data on the left block, the boundary of the left block will be pushed front to *dg* (Lines 13–15, Algorithm 5). On the other hand, on the right block, the boundary of the right block will be pushed back to *dl* (Lines 16–18, Algorithm 5). This iteration will be run until index *i* is equal to *j* and then return *dltemp* and *dgtemp* to run the sequential partitioning function. The *Multi-Deque Partitioning phase* and *Dual-Deque Merging phase* are illustrated in Fig. 1.

### Sorting phase

The parallel sorting algorithm with the divide and conquer concept consists of two parts. The divide step is parallel partitioning, which is the *Multi-Deque Partitioning* and *Dual-Deque Merging phases* in this work. While the data are divided and smaller than the cutoff, the *Sorting phase* (Conquer) is executed in parallel using the **omp task** (Lines 1–4, Algorithm 1).

### Lomuto’s vs. Hoare’s partitioning in the Multi-Deque partitioning phase

The previous phases show the *Multi-Deque Partition Dual-Deque Merge sorting* algorithm. The *Multi-Deque Partitioning phase*, which is an important phase, uses *Hoare’s partitioning* algorithm to partition the data in each block (Lines 11–22, Algorithm 3). In this paper, we compare *Hoare’s* and *Lomuto’s partitioning* algorithms in the *Multi-Deque Partitioning phase* to improve the performance of our *MPDMSort*.

## Experiments, results and discussions

This section shows the experimental setup of the sorting algorithms and compares the results of run time, speedup, speedup comparison between *Lomuto’s* and *Hoare’s partitioning* in the *Multi-Deque Partitioning phase*, and comparison of speedup per thread, which is the efficiency of the algorithm with other related sorting algorithms, and profiles the algorithms with the *Perf profiling tool*.

### Experimental setup

In this paper, the *MPDMSort* is compared with the *Parallel Balanced Quicksort* (*BQSort*), *Multiway Merge Sort* (*MWSort*) and *STLSort* algorithms. Datasets are generated as random, nearly sorted and reversed 32-bit and 64-bit unsigned integers. Data size *n* are 200, 500, 1000, and 2000 million data. The block size *b* values in the *Multi-Deque Partitioning phase* are 0.5 MB, 1 MB, 2 MB, 4 MB, and 8 MB. The *cutoff* values in the *Sorting phase* were 16 MB, 32 MB, and 64 MB. The *MPDMSort*, *BQSort*, *MWSort* and *STLSort* are executed on 2 computers: one with a 16-core Intel Xeon Gold 6142 processor with 64 GB of main memory running Ubuntu Linux on a virtual machine and set the number of cores on virtual machine to 16 cores, and one with an 8-core Intel i7-11700 processor with 16 GB of main memory also running Ubuntu Linux.

### Results

In this paper, run time, speedup, speedup per thread, and the *Perf profiling tool* are used to measure the performance metrics of the algorithms.

#### Run time

**Table 1:** Average Run time of each block size b (Uint32) of random distribution on the Intel Xeon Gold 6142 machine vs. the i7-11700 machine at *cutoff* = 32 MB.

*n* (million)	*b* (MB)	Xeon Gold 6142		i7-11700	
		Run time (s)	STD	Run time (s)	STD
200	0.5	1.8651	0.0765	1.8881	0.0811
	1	1.8680	0.0814	1.5872	0.0492
	2	1.8765	0.0689	1.5811	0.0424
	4	1.8802	0.0696	1.5853	0.0453
	8	1.8716	0.0746	1.5846	0.0459
500	0.5	4.3062	0.0926	4.4356	0.0969
	1	4.3150	0.0835	3.8543	0.0559
	2	4.3360	0.0996	3.8661	0.0565
	4	4.3099	0.0863	3.8759	0.0604
	8	4.3332	0.0901	3.8865	0.0635
1000	0.5	8.4703	0.1313	8.5237	0.1521
	1	8.4514	0.1474	8.2717	0.1139
	2	8.5167	0.1470	8.2314	0.1168
	4	8.5031	0.1414	8.2757	0.1223
	8	8.5112	0.1437	8.2632	0.1059
2000	0.5	17.1971	0.2543	17.3835	0.2628
	1	17.2079	0.2620	18.5480	0.2289
	2	17.1372	0.2846	18.1970	0.2336
	4	17.1842	0.2479	18.1750	0.2087
	8	17.2446	0.2426	18.2226	0.2144

*MPDMSort* is the parallel block-based sorting algorithm. Each thread runs its partitioning algorithm in each block. Therefore, block size *b* is an important parameter that affects the run time of *MPDMSort*. Table 1 shows the average run time of each block size *b* of random distribution on the Intel Gold 6142 machine vs. the i7-11700 machine at *cutoff* = 4.

The best sorting run time is at *b* = 0.5 MB while sorting 200 and 500 million data on the Intel Xeon Gold 6142 machine. While the data are increased to 1000 and 2000 million, the *b* values are increased to 1 and 2 MB, respectively. On the other hand, the *b* values are between 1 MB and 4 MB on the i7-11700 machine. Its *b* values are increased when *n* increases, which is similar to the Intel Xeon Gold 6142 machine.

*MPDMSort* is developed by the divide and conquer algorithm concept. Therefore, it needs to switch the divide part into the conquer part. The parameter used to switch to the conquer part is the *cutoff*. Table 2 shows the average run time of each *cutoff* of random distribution on the Intel Xeon Gold 6142 machine vs. the i7-11700 machine at *b* = 1 MB.

**Table 2:** Average run time of each cutoff (Uint32) of random distribution on the Intel Xeon Gold 6142 machine vs. the i7-11700 machine at *b* = 1 MB.

*n* (million)	Cutoff (MB)	Xeon Gold 6142		i7-11700	
		Run time (s)	STD	Run time (s)	STD
200	16	1.7262	0.0418	1.4951	0.0286
	32	1.8680	0.0814	1.5872	0.0492
	64	2.1410	0.1505	1.7377	0.0976
500	16	4.2012	0.0752	3.8609	0.0583
	32	4.3150	0.0835	3.8543	0.0559
	64	4.6471	0.1584	4.0321	0.0951
1000	16	8.4409	0.1283	8.4288	0.1057
	32	8.4514	0.1474	8.2717	0.1139
	64	8.7463	0.1892	8.2365	0.1103
2000	16	17.2535	0.2582	18.5480	0.2574
	32	17.2079	0.2620	18.1864	0.2289
	64	17.3835	0.2791	17.9934	0.2112

The best run time values in each *n* of *MPDMSort* are between 16 MB and 32 MB. Note that the *cutoff* is 32 MB at *n* = 2000 million data on the Intel Xeon Gold 6142 machine. It can be noticed that most of the run time of the smaller *cutoff* is more stable than that of the larger *cutoff*. On the other hand, the Intel i7-11700 machine’s best run time values are increased from 16 MB to 64 MB. Its *cutoff* is proportional to *n*.

Table 3 shows the average run time of random, reversed, nearly sorted distributions for sorting Uint64 200, 500, 1000, and 2000 million data on the Intel Xeon Gold 6142 machine at *b* = 2 MB and *cutoff* = 32 MB.

*BQSort* is the fastest algorithm while sorting the random distribution at *n* = 200 and 500 million data. However, *MPDMSort* can sort the random data faster than *BQSort* and *MWSort* at 1000 and 2000 million data. On the other hand, *MWSort* and *BQSort* are the fastest when sorting the reversed and nearly sorted distributions, respectively. This means that our *MPDMSort* can sort the larger random distribution data better than the other sorting algorithms. This is the limitations of *MPDMSort*. The first limitation is sorting the reversed and nearly sorted distributions. It can be noticed that run time of *BQSort* is the best while sorting nearly sorted distribution and *MWSort* run time is the best for reversed distribution. This can be dued to the *Multi-Deque Partitioning phase* is the block-based partitioning concept which uses the *Hoare’s* partitioning in each block. However, this concept uses compare-and-swap operation in each block every level of the subarray. This consumes run time greater than the Multiway-Merge algorithm in the *MWSort*. The second limitation of *MPDMSort* is sorting the small random distribution data. We can notice that run time of *BQSort* is the best while sorting smaller data such as 200 and 500 million random data. This can be dued to the overhead of our sequential region of *MPDMSort*, for example *Dual-Deque Merging Phase* and critical section in the parallel region such as push and pop operations of deques. Moreover, the average run time of two machines are very similar. It can be dued to the number of cores on virtual machine of Intel Xeon Gold 6152 are set to 16 cores. There are 16 hardware threads which are equal to Intel Core i7-11700 machine.

**Table 3:** Average Run time of each distribution for sorting Uint64 data on the Intel Xeon Gold 6142 machine (NSorted: Nearly Sorted, M: Million) at *b* = 2 MB and *cutoff* = 32 MB.

Algorithms	Dist.	Run time (s)			
		*n*=200 M	*n* = 500 M	*n* = 1000 M	*n* = 2000 M
*MPDMSort*	Random	1.8765	4.3360	8.5167	17.1372
	Reversed	0.7904	2.0178	4.0962	8.5175
	NSorted	1.5656	3.8825	7.9081	16.3950
*BQSort*	Random	1.6735	4.3180	8.9265	18.4044
	Reversed	1.2896	3.3464	6.8680	14.4659
	NSorted	1.4478	3.7744	7.8678	15.7210
*MWSort*	Random	1.7522	4.5322	9.2801	19.5174
	Reversed	0.7225	1.8141	3.7310	7.7516
	NSorted	1.5889	4.0359	17.1523	17.1523
*STLSort*	Random	19.6084	51.4723	106.5146	220.4606
	Reversed	4.3735	11.6869	24.6071	51.6390
	NSorted	18.0887	47.1532	99.1747	202.2801

#### Speedup

**Figure 2:**
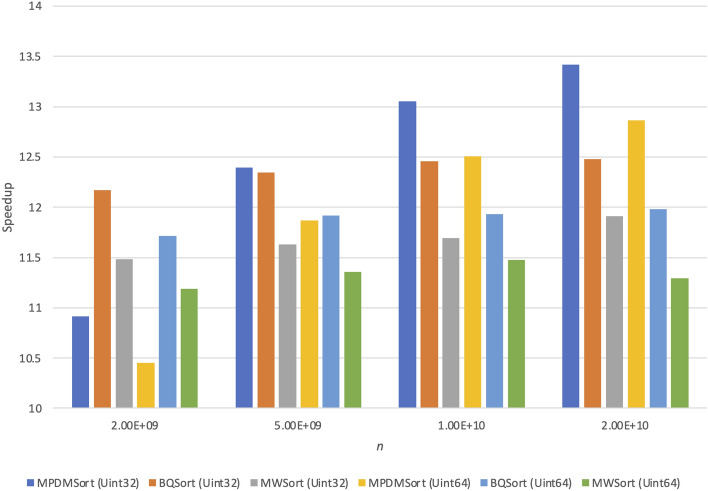
Speedup of any parallel sorting algorithm (random) on the Intel Xeon Gold 6142 machine.

In this paper, we measure the run time metric of each algorithm and calculate them using the run time of *STLSort* and the run time of *MPDMSort*, *BQSort*, and *MWSort*. Figure 2 shows the speedup of any parallel sorting algorithm with a random distribution on the Intel Xeon Gold 6142 machine. The speedup metrics of all algorithms are proportional to *n*. The best speedups of *MPDMSort* and *BQSort* are at *n*=2000 million data. However, the best speedup of *MWSort* is at *n*=1000 million data. The Speedup of our *MPDMSort* is proportional to the input size. It can be due to the overhead of the OpenMP library and fraction between the sequential and parallel regions of the parallel algorithm.

**Figure 3:**
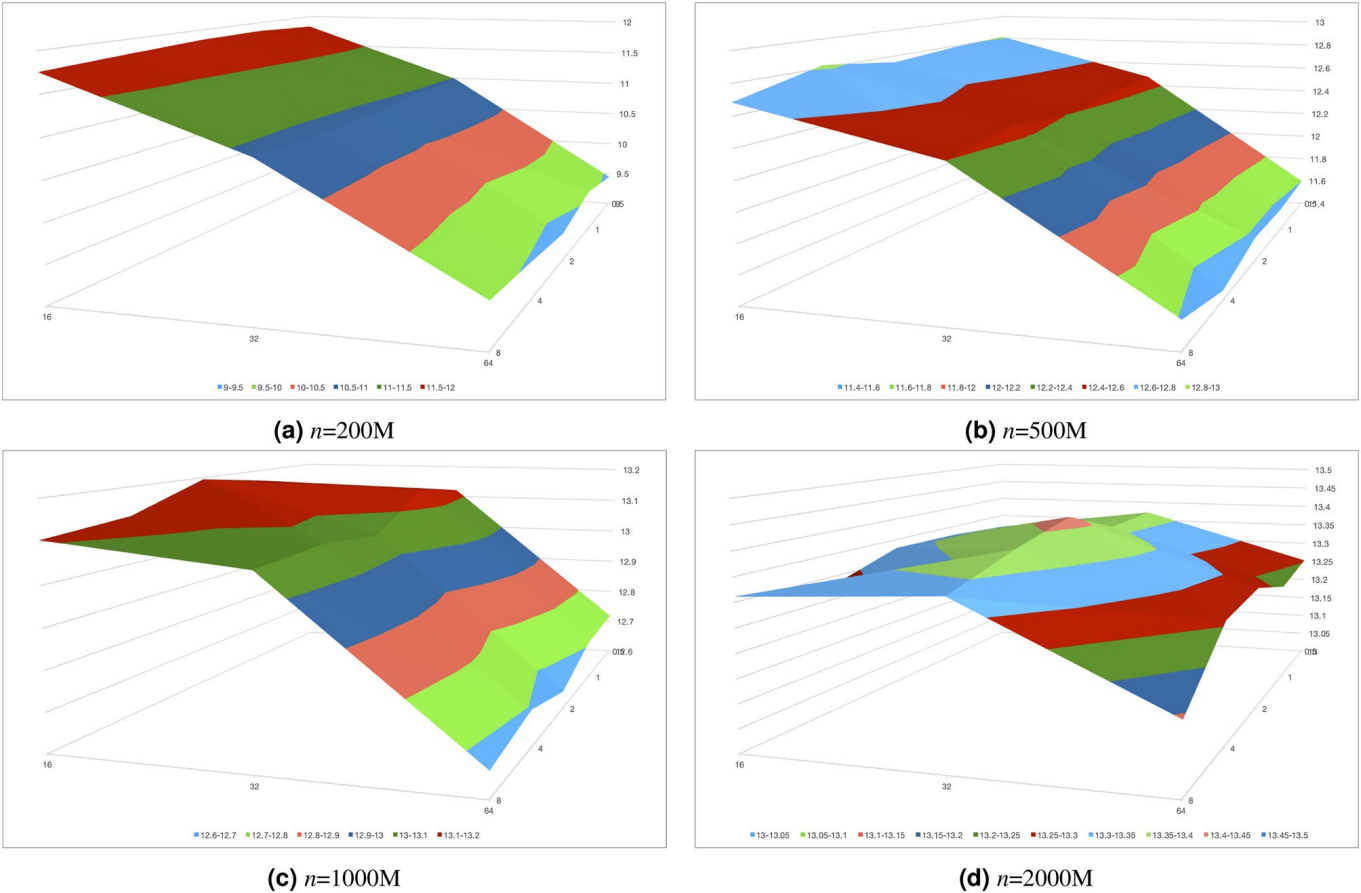
Speedup vs *b* vs *cutoff* of *MPDMSort* sorts random Uint32 data on the Intel Xeon Gold 6142 machine (M: Million).

Figure 3 shows speedup of *MPDMSort* vs *b* vs *cutoff* sortss random Uint32 data on the Intel Xeon Gold 6142 machine. We can notice that the best speedup of *n* = 200 M which is small dataset as shown in Fig. 3a is at *b* = 1 and *cutoff* = 16 MB. When *n* = 500 M and 1000 M, *b* is increased between 2 and 4 with *cutoff* = 16 MB as shown in Fig. 3b and c. Figure 3d shows speedup of *MPDMSort* vs *b* vs *cutoff* at *n*=2000 million data where *b* = 0.5, 1, 2, 4, 8 MB and *cutoff* = 16, 32, 64 MB. The best speedup is up to 13.42$\times$ at *b* = 2 and *cutoff* = 32 MB. It can be noticed that *n* is increased, *b* grows from 1 to 2. After that, *cutoff* is incresed from 16 to 32 while *n*=2000 million data. *b*=2 and *cutoff*=32 are the set of parameters which can be choosen to sort the data by our *MPDMSort*.

#### Lomuto’s vs. Hoare’s partitioning in Multi-Deque partitioning results

Table 4 shows the best speedup of *MPDMSort* with *Lomuto’s* vs. *Hoare’s partitioning* in the *Multi-Deque Partitioning phase*. We note that Speedups of *Lomuto’s partitioning* in our algorithm are greater than *Hoare’s* in all parameters. This can be due to the block size of our work. Our algorithm uses a block-based parallel partitioning concept in which every block is small and residents the cache. The indices of *Lomuto’s partitioning* are increased and run from left to right. However, *Hoare’s partitioning* uses two indices. The first index runs from left to right, and the second index runs from right to left, and its locality is not better than that of *Lomuto’s partitioning* algorithm.

**Table 4:** Best speedup of MPDMSort with Lomuto’s vs. Hoare’s partitioning in Multi-Deque Partitioning results on the Intel Xeon Gold 6142 machine.

*n*(million)	Speedup			
	Uint32		Uint64	
	Lomuto’s	Hoare’s	Lomuto’s	Hoare’s
200	12.24	11.87	11.75	11.36
500	13.24	12.82	12.76	12.28
1000	13.59	13.20	13.07	12.65
2000	13.81	13.42	13.29	12.86

#### Speedup per thread

Speedup per thread is the metric that can be used to measure the performance of the parallel algorithm. When Speedup per thread is greater, the processor core can be used efficiently. Speedup per thread is the fraction of Speedup of the algorithm and hardware threads in any processor. We can use this metric to compare the parallel algorithms because this metric cannot be greater than 1.00.

**Table 5:** Best Speedup per thread of each parallel sorting algorithm.

Algorithms	Speedup	Processor cores	Hardware thread	Speedup/thread
$MPDMSort_{Lomuto}$	13.81	16	16	0.86
$MPDMSort_{Hoare}$	13.42	16	16	0.84
*BQSort*	12.48	16	16	0.78
*MWSort*	11.48	16	16	0.72
*MultiSort*^21^	13.60	32	32	0.43
*psort*^22^	11.00	24	24	0.46
*Introqsort*^23^	1.47	2	2	0.74
*PPMQSort*^25^	12.29	8	16	0.77
*HDPSort*^26^	2.49	4	8	0.31
*CPP*11*Sort*^32^	44.2	56	56	0.79
$DPPSort_{STL}$^33^	5.88	4	8	0.74

Table 5 shows the best speedup per thread of each parallel sorting algorithm. Note that *MPDMSort*, *BQSort*, and *MWSort* are run on the same machine in this experiment. We note that Speedup per thread of both $MPDMSort_{Lomuto}$ and $MPDMSort_{Hoare}$ are greater than the others. The Speedup per thread of all parallel sorting algorithms in this table, such as *BQSort* (0.78), *MWSort* (0.72), *MultiSort*^21^ (0.43), *psort*^22^ (0.46), *Introqsort*^23^ (0.74), *PPMQSort*^25^ (0.77), *HDPSort*^26^ (0.31), *CPP*11*Sort*^32^ (0.79) and $DPPSort_{STL}$^33^ (0.74), are smaller than those of our $MPDMSort_{Lomuto}$ and $MPDMSort_{Hoare}$. This metric shows the performance of our parallel partitioning algorithm, which uses the block-based concept, and our merging algorithm without the compare-and-swap operation.

$MPDMSort_{Lomuto}$ uses the block-based partitioning concept in *Multi-Deque partitioning* phase and implements *Lomuto’s* partitioning inside. It improves the locality of partitioning algorithm while it executes in parallel. This technique is used in the algorithms with high Speedup per thread metric such as *BQSort*, *Introqsort*, *PPMQSort*, *CPP*11*Sort*, and $DPPSort_{STL}$. Note that, *PPMQSort* and $DPPSort_{STL}$ begins with 2 blocks in parallel partitioning phase. Moreover, most of block-based partitioninig algorithms merge the unpartitioned data by moving them to the middle. Then, partitioning them in sequential or paralell which consumes compare-and-swap operations that affects run time. However, our $MPDMSort_{Lomuto}$ uses the *Dual-Deque Merging* phase to merge the partitioned data in each block which reduces compare-and-swap operations. Therefore, speedup per thread of $MPDMSort_{Lomuto}$ is greater than the other algorithms.

#### Perf Profiling tool

The *Perf profiling tool* is the Linux tool^13^ that can profile the metrics that affect the performance of the sorting algorithm. $MPDMSort_{Lomuto}$, $MPDMSort_{Hoare}$, *BQSort* and *MWSort* are run to sort the data and profile the metrics. Table 6 shows the *Perf* results of four parallel algorithms on the Intel i7-11700 machine.

**Table 6:** Perf results of $MPDMSort_{Lomuto}$, $MPDMSort_{Hoare}$, *BQSort*, and *MWSort* on the Intel i7-11700 machine at *n* = 1000 million Uint64 data.

Algorithms	*b*	Cutoff	Cache misses	Branch Load misses
$MPDMSort_{Lomuto}$	2 MB	16 MB	2.73E+09	1.09E+10
		32 MB	2.64E+09	1.18E+10
		64 MB	2.69E+09	1.19E+10
	4 MB	16 MB	2.84E+09	1.12E+10
		32 MB	2.86E+09	1.14E+10
		64 MB	2.69E+09	1.23E+10
	8 MB	16 MB	3.07E+09	1.12E+10
		32 MB	2.99E+09	1.16E+10
		64 MB	2.92E+09	1.20E+10
$MPDMSort_{Hoare}$	2 MB	16 MB	2.40E+09	1.13E+10
		32 MB	2.52E+09	1.14E+10
		64 MB	2.42E+09	1.20E+10
	4 MB	16 MB	2.46E+09	1.09E+10
		32 MB	2.47E+09	1.14E+10
		64 MB	2.57E+09	1.16E+10
	8 MB	16 MB	2.58E+09	1.09E+10
		32 MB	2.52E+09	1.13E+10
		64 MB	2.43E+09	1.20E+10
*BQSort*			2.12E+09	1.26E+10
*MWSort*			1.90E+09	8.61E+09

In this experiment, we increase *b* into 2 MB, 4 MB and 8 MB to show the effect of block size in each algorithm. Note that the *cutoff* values are set to 16 MB, 32 MB and 64 MB. The important metrics that affect the run time of any parallel sorting algorithm are branch load misses and cache misses^33^.

We note that *b* is proportional to cache misses. If *b* is increased, cache misses are increased. Moreover, the *cutoff* is proportional to the branch load misses value. If the *cutoff* value is increased, the branch load misses value is increased.

## Conclusions

In this paper, the *Multi-Deque Partition Dual-Deque Merge sorting* algorithm, which is a parallel block-based sorting algorithm on a shared-memory system, is proposed. Its concept of *MPDMSort* is to partition in the *Multi-Deque Partitioning phase*. The *Hoare’s* and *Lomuto’s* partitioning are compared in this phase and found that *Lomuto’s* partitioning is faster than the *Hoare’s* partitioning algorithm. Then, the partitioning result is merged in the *Dual-Deque Merging phase*. The *Dual-Deque Merging phase* is the main contribution of this work which reduces compare-and-swap operations which can solve the problem of parallel block-based partitioning. This parallel algorithm executes recursively until the data are smaller than the *cutoff* parameter. Finally, the partitioned data are sorted independently using *STLSort*, which is a sequential standard sorting function.

*MPDMSort* is implemented and executed on 2 computers: one with an Intel Xeon Gold 6142 processor running Ubuntu Linux, and the other with an Intel Core i7-11700 processor also running Ubuntu Linux. The *MPDMSort* run time is faster than those of *BQSort* and *MWSort*. Its speedup of 13.81$\times$ and speedup per thread of 0.86 can be obtained while sorting random distribution data. Speedup per thread of *MPDMSort* is greater than the other paralell sorting algorithms. Its speedup of *MPDMSort* depends on the block size, cutoff, data size, type, and distribution. The important metrics which affect the performance of parallel sorting algorithm are cache misses that is proportional to *b* and branch load misses that is proportional to *cutoff*. This method can be used to merge the data in the paralell algorithm with block-based concept.

## Data Availability

In this paper, we use the datasets generator which can generate random, reversed, and nearly sorted 32-bit and 64-bit data. The *newinitData()* can be called after it is implemented in the source code. Then, it will generate the datasets in the array which is passed into the *newinitData()*. The source code of datasets generator during the current study are available in the DataSetGenerator repository, https://www.github.com/apisitjoe/DataSetGenerator or request from the corresponding author, Apisit Rattanatranurak.
